# Supplementary material for: Reconciling Mining with the Conservation of Cave Biodiversity: A Quantitative Baseline to Help Establish Conservation Priorities
Source: PLoS One. 2016 Dec 20;11(12):e0168348. doi: 10.1371/journal.pone.0168348 (PMC5173368; doi:10.1371/journal.pone.0168348)
Supplement: S1 Dataset — (ZIP) [file pone.0168348.s002.zip › Taxa/Serra Norte/SN_2013/N4E/Lista N4E-70.pdf]

CAVIDADE N4E-0070

Classe	Ordem	Fam/Outros	Gên/Outros	Espécie	S-U	U-S	Zon.
Annelida	Oligochaeta			jovem		1	E
Arachnida	Acari	(Parasitengonina)		sp.2		X	E
Arachnida	Acari	Dolichocybidae		sp.1		X	E
Arachnida	Acari	Trombiculidae		sp.39		X	E
Arachnida	Acari	Veigaiidae		sp.1		X	P
Arachnida	Amblypygi	Charinidae	*Charinus*	sp.1		1	E
Arachnida	Amblypygi	Phrynidae	*Heterophrynus*	*longicornis*	3	1	E/P
Arachnida	Araneae	Araneidae	*Alpaida*	aff. *negro*	X	X	E
Arachnida	Araneae	Barychelidae		jovem	1		E
Arachnida	Araneae	Corinnidae		jovem		5	E
Arachnida	Araneae	Ctenidae		jovem	3		E
Arachnida	Araneae	Dipluridae	*Linothele*	sp.1	2		E
Arachnida	Araneae	Drymusidae		jovem		X	E/P
Arachnida	Araneae	Filistatidae		sp.1	X	X	E
Arachnida	Araneae	Mysmenidae	*Microdipoena*	sp.1		X	E
Arachnida	Araneae	Ochyroceratidae	*Ochyrocera*	sp.2		X	E
Arachnida	Araneae	Ochyroceratidae	*Ochyrocera*	sp.3	X	X	E
Arachnida	Araneae	Ochyroceratidae	*Ochyrocera*	sp.4	X		E
Arachnida	Araneae	Ochyroceratidae	*Speocera*	sp.1		X	E/P
Arachnida	Araneae	Pholcidae	*Leptopholcus*	sp.1	X		E
Arachnida	Araneae	Pholcidae	*Mesabolivar*	*aurantiacus*	X		E
Arachnida	Araneae	Pholcidae	*Mesabolivar*	sp.1	X		E
Arachnida	Araneae	Pholcidae		jovem	X	X	E/P
Arachnida	Araneae	Salticidae		sp.4		X	E
Arachnida	Araneae	Scytodidae	*Scytodes*	*eleonorae*	2	5	E
Arachnida	Araneae	Segestriidae		jovem		X	E
Arachnida	Araneae	Theraphosidae	aff. *Holothele*	sp.1	11	1	E
Arachnida	Araneae	Theridiidae	*Theridion*	sp.1	X	X	E
Arachnida	Araneae	Theridiosomatidae	*Plato*	sp.1	X	X	E/P
Arachnida	Araneae	Uloboridae	*Uloborus*	sp.1	X		E
Arachnida	Opiliones	Sclerosomatidae	*Prionostema*	sp.1	7	3	E
Arachnida	Pseudoscorpiones	Chthoniidae	*Pseudochthonius*	sp.1		X	E
Arachnida	Schizomida	Hubbardiidae	aff. *Rowlandius*	sp.1		X	E
Chilopoda	Geophilomorpha	Mecistocephalidae	*Mecistocephalus*	sp.1		1	E
Chilopoda	Scolopendromorpha	Cryptopidae	*Cryptops*	jovem		1	E
Chilopoda	Scolopendromorpha	Scolopocryptopidae	*Scolopocryptops*	sp.1		1	E
Chilopoda	Scolopendromorpha	Scolopocryptopidae	*Tidops*	sp.1		2	E
Chilopoda	Scolopendromorpha			jovem	1		E
Chilopoda	Scutigeromorpha	Pselliodidae		jovem		1	P
Diplopoda	Polydesmida	Chelodesmidae		jovem	1	1	E
Diplopoda	Polydesmida	Cyrtodesmidae		jovem	X		E
Diplopoda	Polydesmida	Fuhrmannodesmidae		sp.1		X	P
Diplopoda	Polydesmida	Pyrgodesmidae		sp.1	X	X	E/P
Diplopoda	Spirostreptida	Pseudonannolenidae	*Pseudonannolene*	sp.2	1		E
Entognatha	Collembola	Entomobryidae		sp.1		X	E
Entognatha	Collembola	Entomobryidae		sp.6	X		E
Entognatha	Collembola	Isotomidae		sp.1		X	E
Entognatha	Collembola	Paronellidae		sp.1		X	E
Entognatha	Collembola	Paronellidae		sp.4		X	P

Entognatha	Diplura	Campodeidae		sp.1	X	X	E
Insecta	Archaeognatha	Meinertellidae		sp.1		X	E
Insecta	Blattodea	Blattellidae		jovem	1	5	E
Insecta	Blattodea	Blattidae		sp.5	3		E
Insecta	Blattodea	Polyphagidae		jovem		5	E
Insecta	Coleoptera	Chrysomelidae		sp.10	X		E
Insecta	Coleoptera	Chrysomelidae		sp.16		X	E
Insecta	Coleoptera	Staphylinidae	Pselaphinae	sp.23		X	E
Insecta	Coleoptera	Staphylinidae	Pselaphinae	sp.29		X	E
Insecta	Coleoptera	Staphylinidae		sp.42	X		E
Insecta	Diptera	Chironomidae		sp.	X		E
Insecta	Diptera	Culicidae	*Culex*	sp.	X		E
Insecta	Diptera	Phoridae		sp.	X	X	E
Insecta	Diptera	Psychodidae	Phlebotominae	sp.	X	X	E
Insecta	Diptera	Sciaridae		sp.		X	E
Insecta	Diptera	Tipulidae	Tipulinae	sp.	X	X	E
Insecta	Heteroptera	Cydnidae		sp.		X	E
Insecta	Heteroptera	Dipsocoroidea		sp.	X	X	E
Insecta	Heteroptera	Lygaeidae		jovem		X	E
Insecta	Heteroptera	Reduviidae	Emesinae	sp.		1	E
Insecta	Heteroptera	Reduviidae	Reduviinae	sp.	4	3	E
Insecta	Homoptera	Cixiidae		sp.4		X	E
Insecta	Hymenoptera	Formicidae	*Camponotus*	sp.1	X	X	E
Insecta	Hymenoptera	Formicidae	*Camponotus*	sp.2		X	E
Insecta	Hymenoptera	Formicidae	*Pachycondyla*	sp.1	X	X	E
Insecta	Hymenoptera	Formicidae	*Pachycondyla*	sp.2	X	X	E/P
Insecta	Hymenoptera	Formicidae	*Pheidole*	sp.2		X	E
Insecta	Hymenoptera	Formicidae	*Trachymyrmex*	sp.1	X		E
Insecta	Hymenoptera	Vespidae		sp.4		X	E
Insecta	Hymenoptera	Vespidae		sp.5		X	E
Insecta	Isoptera	Rhinotermitidae	*Heterotermes*	sp.		X	E
Insecta	Isoptera	Termitidae	*Nasutitermes*	sp.	X	X	E
Insecta	Lepidoptera	Gracillariidae		sp.		X	E
Insecta	Lepidoptera	Noctuoidea		sp.1	1	1	E
Insecta	Lepidoptera	Noctuoidea		sp.4	1		E
Insecta	Lepidoptera	Noctuoidea		sp.5		2	E
Insecta	Lepidoptera	Roeslerstammiidae		sp.		X	E
Insecta	Lepidoptera	Tineoidea		sp.1		X	E
Insecta	Lepidoptera	Tineoidea		sp.2	X	X	E
Insecta	Orthoptera	Phalangopsidae	*Paraclodes*	sp.	25	9	E
Insecta	Orthoptera	Phalangopsidae	*Phalangopsis*	sp.	33	80	E/P
Insecta	Psocoptera	Archipsocidae	*Archipsocus*	sp.1	X		E
Insecta	Psocoptera	Epipsocidae	*Epipsocus*	sp.2	X		E
Insecta	Psocoptera	Psyllipsocidae	*Psyllipsocus*	sp.1		X	E
Insecta	Psocoptera	Ptiloneuridae	*Triplocania*	sp.1		X	E
Insecta	Siphonaptera	Leptopsyllidae		sp.1		X	E
Insecta	Thysanura	Nicoletiidae		sp.1		X	E
Gastropoda	Pulmonata	Bulimulidae	*Naesiotus*	sp.		X	E
Gastropoda	Pulmonata	Systrophiidae	*Happia*	sp.	X		E
Malacostraca	Isopoda	Armadillidae		sp.1		X	E
Malacostraca	Isopoda	Dubioniscidae		sp.1		X	E
Malacostraca	Isopoda	Philosciidae		sp.1	X	X	E

Malacostraca	Isopoda	Platyarthridae		sp.1	X	X	E
Malacostraca	Isopoda	Scleropactidae		sp.1		X	P
Amphibia	Anura	Strabomantidae	*Pristimantis*	cf. *fenestratus*	6	4	E
Mammalia	Chiroptera	Emballonuridae	*Peropteryx*	*kappleri*	20	15	E/P
Mammalia	Chiroptera	Furipteridae	*Furipterus*	*horrens*		4	E/P
Mammalia	Chiroptera	Phyllostomidae	*Carollia*	*perspicillata*		10	E/P
Mammalia	Chiroptera	Phyllostomidae	*Glossophaga*	*soricina*	1		E/P
Mammalia	Chiroptera	Phyllostomidae	*Trachops*	*cirrhosus*	3		E/P
